# Segmental structure in banded mongoose calls

**DOI:** 10.1186/1741-7007-10-98

**Published:** 2012-12-03

**Authors:** W Tecumseh Fitch

**Affiliations:** 1 Department of Cognitive Biology, University of Vienna, 14 Althanstrasse, A-1090 Vienna, Austria

## Abstract

In complex animal vocalizations, such as bird or whale song, a great variety of songs can be produced via rearrangements of a smaller set of 'syllables', known as 'phonological syntax' or 'phonocoding' However, food or alarm calls, which function as referential signals, were previously thought to lack such combinatorial structure. A new study of calls in the banded mongoose *Mungos mungo *provides the first evidence of phonocoding at the level of single calls. The first portion of the call provides cues to the identity of the caller, and the second part encodes its current activity. This provides the first example known in animals of something akin to the consonants and vowels of human speech.

See research article http://www.biomedcentral.com/1741-7007/10/97

## 

Studies of animal communication often start by comparing the structure and function of animal vocalizations to human language. But some comparisons are more revealing than others. For example, a superficial similarity is that animal vocalizations are acoustic signals, like human speech. But since language can be readily expressed in non-acoustic domains, such as writing or sign language, this similarity provides little insight into other core aspects of language, such as its capacity to produce an unlimited range of meaningful sentences - what linguist Wilhelm von Humboldt called 'making infinite use of finite means' [1].

Language achieves its remarkable productive capacity by combining small 'atomic' units called 'phonemes' or 'segments' into hierarchically structured syllables and words, words into phrases and sentences, and sentences into stories and conversations. This combinatoric productivity thus occurs at multiple levels. The most obvious level is the combination of meaningful words into sentences, whose meaning is a complex composition of those words - the level of phrasal syntax. Another level involves combining meaningless phonemes into syllables and syllables into words, termed 'phonological syntax'. Linguist Charles Hockett dubbed this two-level combinatorial principle of language 'duality of patterning' [2].

Why does this duality exist? Although there are an unlimited number of sentences in any language, the number of distinctive sounds that we can produce and discriminate is limited, constrained both by the perceptual difficulty of hearing fine differences, and the motoric difficulty of producing them. All languages therefore make use of a relatively small set of discrete vowels and consonants to build up larger linguistic entities. Languages vary considerably in the number of segments they use. For example, the Hawaiian language uses a set of only five vowels and eight consonants. But this small set nonetheless permits 40 different consonant-vowel syllables, from which 40^5 ^= 100 million different five-syllable words could be constructed (or 78 million if no syllable is repeated). The combinatoric power of this approach is obvious: a small number of atomic acoustic units, each of which is easily produced and discriminated, yields a practically unlimited number of possible words.

Phonological syntax also involves clear rules. In English, for example, the 'ng' phoneme that appears at the end of 'sing' or 'rang' can only come at the end of a syllable, not the beginning. Thus, although it uses the same segments as 'rang', 'ngar' is not a phonologically permitted word in English (though it would be acceptable in some other languages, or perhaps in English on 'Talk Like a Pirate Day').

To what extent are such combinatorial principles utilized in animal communication?

Ethologist Peter Marler introduced the terms 'lexicoding' for sentence-building and 'phonocoding' for word building [3]. Marler suggested that only phonocoding provides an appropriate comparison for animal communication systems like bird or whale song, and most birdsong researchers and linguists today agree [4,5]. Finally, Marler observed that all the known examples of meaningful alarm calls and food calls 'come as an indivisible package', showing no evidence of phonocoding.

The basic unit of phonological syntax in many bird and primate songs is termed a 'syllable', defined as an uninterrupted trace in a spectrographic signal. Considerable research has shown how such syllables can be combined into more complex phrases and songs [4]. Many bird species follow quite strict rules of combination. For example, the call for which the black-capped chickadee (*Poecile atricapillus*) is named follows a strict order: a variable number of 'chicks' always precede a number of 'dees' [6]. Furthermore, when this call signals the presence of a predator, the number of 'dees' correlates with the predator's size and dangerousness [7].

It is tempting to think of chickadee syllables as playing a role akin to segments in human speech. The difficulty with this analogy is that each chickadee syllable is a discrete expiratory chunk, with each syllable separated by a moment of silence. In contrast, in a word like 'string,' five different segments are combined in a single continuously pronounced syllable. There is little prior evidence that animals combine discrete segmental units in a single-syllable call in this manner, despite abundant evidence for sequential combinations of syllables into phrases. Enter the banded mongoose, *Mungos mungo*, whose contact calls appear to possess just such segmental structure, as documented in this issue of *BMC Biology *in a paper by David Jansen and colleagues [8].

Animal communication research typically focuses on a restricted set of taxa, especially frogs, songbirds, and primates. Recently, however, small carnivores in the mongoose family have started attracting attention. A series of publications on meerkats *Suricata suricatta *by Marta Manser (senior author of the Jansen paper) show that these iconic African mammals produce functionally referential alarm calls (Figure 1a). Meerkat alarm calls signal both predator class (terrestrial versus aerial predator) and degree of threat (how close the predator is) [9]. The richness of the meerkat vocal system has inspired research on other members of the mongoose family (Herpestidae). Herpestids are widespread in Africa and southeast Asia, occupying a wide array of ecological niches, and have social systems ranging from solitary to highly social cooperative breeders. This diversity, and the fact that many species are highly vocal, makes mongooses an excellent taxon for studying the evolution of communication.

**Figure 1:**
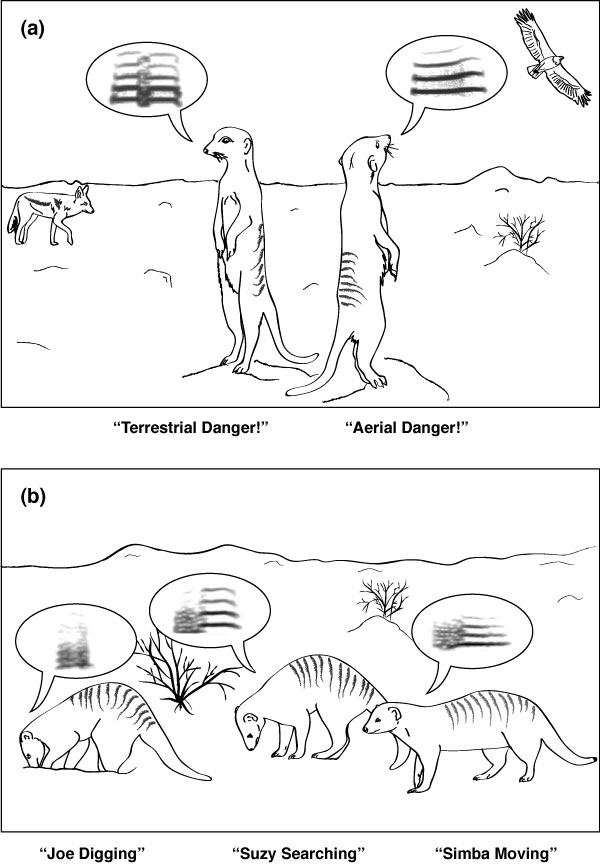
**Schematic of vocalization types in members of the mongoose family**. **(a) **Meerkats *Suricata suricatta *produce functionally referential alarm calls that signal both predator location (terrestrial versus aerial) and urgency. **(b) **Banded mongooses (*Mungos mungo*) produce close calls that sequentially encode both individual identity (a 'vocal signature') and current activity. This provides the first known example in animals of phonological syntax within a single referential signal. (Figure by Nadja Kavcik.)

Banded mongooses are social carnivores that live in African forests and grasslands in groups of around 20 animals. Group members defend their territory, keep watch, and breed cooperatively. They are highly vocal, and because they spend considerable time digging, with their heads down, vocal cues from other group members play an important role (Figure 1b). Banded mongooses produce different 'close calls' during different activities (searching, moving, digging, and so on), and previous playback experiments with this species showed that both pups and adults can recognize other individuals from their calls [10]. The close calls themselves are continuous and quite short, from 50 to 150 ms, and are thus comparable to a single spoken syllable. An initial noisy portion resembles a noisy consonant like 's' or 't', while the second tonal portion is more vowel-like. These results provided the starting point for the acoustic analysis reported in this issue.

Jansen and colleagues used a permuted discriminant function analysis to analyze close calls for cues to individuality (controlling for behavioral context) and behavioral context (controlling for individuality). They found that calls could be accurately categorized at both levels. Furthermore the initial noisy segment carried most of the information required to recognize the individual calling. The second more tonal segment of the call, when present, carries all of the information indicating the caller's activity. Changing either component changes the composite meaning in specific ways, just as 'Jay' is different from both 'Kay' and 'Joe'. This study provides the first clear example of segmental encoding, akin to the consonants and vowels of speech, within a single animal 'syllable' or call.

Banded mongoose close calls resemble the syllabic coding of speech more than the well-documented phonocoding in birds or whales, where atomic syllables are combined into larger phrases and songs. They also differ from the many examples from primates and other taxa whose calls are individually distinctive ('acoustic signatures'), because in such cases the individual and contextual information is fused together in a syllable, rather than sequentially arranged. That this is the first demonstration of segmental concatenation in animal communication does not necessarily mean that this is rare: Jansen and colleagues speculate that segmental encoding may be present, but overlooked, in other species. The methods introduced here will provide a useful tool for similar investigations of other species.

Many questions about the intriguing call system of the banded mongoose remain unanswered, and an extensive set of playback experiments will be needed to determine what, exactly, listeners extract from these calls. Can listeners recognize unfamiliar callers as individuals from their calls? How are the two classes of information acquired during development? Do pups start out with a single all-purpose syllable and differentiate it into the initial and terminal segments? Or do they start with separate segments and only later fuse them into one syllable? In speech, adjacent segments affect each other via 'co-articulation'. Thus, the vowel in 'pop' is subtly different from that in 'not', because the latter is nasalized by the presence of the initial 'n'. Is such co-articulation present in banded mongoose calls?

This research offers a refreshing new perspective for comparisons of human language and animal vocal communication. Humans are understandably interested in the most complex aspects of language, and ethologists are thus often tempted to draw analogies with sophisticated linguistic features like phrasal syntax and semantics. Perhaps by doing so we have overlooked the considerable complexity and sophistication that may lie within a single call or syllable, where linguistic phonology offers a more apt comparison.

Ultimately, of course, animal signals need to be interpreted not just in comparison to human language, but in their own terms, relative to the ecology and social structure of the species in question. For such questions, we can expect further investigations of communication in banded mongooses, and other mongoose species, to yield rich insights into the evolution of communication.
